# Copper and Zinc Particles as Regulators of Cardiovascular System Function—A Review

**DOI:** 10.3390/nu15133040

**Published:** 2023-07-05

**Authors:** Klaudia Kitala, Damian Tanski, Janusz Godlewski, Magdalena Krajewska-Włodarczyk, Leszek Gromadziński, Michał Majewski

**Affiliations:** 1 Department of Pharmacology and Toxicology, Faculty of Medicine, University of Warmia and Mazury in Olsztyn, 10-082 Olsztyn, Poland; michal.majewski@uwm.edu.pl; 2 Department of Human Histology and Embryology, Faculty of Medicine, University of Warmia and Mazury in Olsztyn, 10-082 Olsztyn, Poland; damian.tanski@uwm.edu.pl (D.T.); janusz.godlewski@uwm.edu.pl (J.G.); 3 Department of Mental and Psychosomatic Diseases, Faculty of Medicine, University of Warmia and Mazury in Olsztyn, 10-082 Olsztyn, Poland; magdalenakw@op.pl; 4 Department of Cardiology and Internal Medicine, Faculty of Medicine, University of Warmia and Mazury in Olsztyn, 10-082 Olsztyn, Poland; leszek.gromadzinski@uwm.edu.pl

**Keywords:** cardiovascular system, copper, nanoparticles, zinc

## Abstract

Copper and zinc are micronutrients that play a crucial role in many cellular pathways, act as cofactors in enzymatic systems, and hence, modulate enzyme activity. The regulation of these elements in homeostasis is precisely controlled by various mechanisms. Superoxide dismutase (SOD) is an enzyme requiring both copper and zinc for proper functioning. Additionally, there is an interaction between the concentrations of copper and zinc. Dietary ingestion of large amounts of zinc augments intestinal absorption of this trace element, resulting in copper deficiency secondary to zinc excess. The presence of an overabundance of copper and zinc has a detrimental impact on the cardiovascular system; however, the impact on vascular contractility varies. Copper plays a role in the modulation of vascular remodeling in the cardiac tissue, and the phenomenon of cuproptosis has been linked to the pathogenesis of coronary artery disease. The presence of copper has an observable effect on the vasorelaxation mediated by nitric oxide. The maintenance of proper levels of zinc within an organism influences SOD and is essential in the pathogenesis of myocardial ischemia/reperfusion injury. Recently, the effects of metal nanoparticles have been investigated due to their unique characteristics. On the other hand, dietary introduction of metal nanoparticles may result in vascular dysfunction, oxidative stress, and cellular DNA damage. Copper and zinc intake affect cardiovascular function, but more research is needed.

## 1. General Characteristics of the Role of Copper and Zinc in Physiological Processes

Adequate amounts of micronutrients in the diets of mammals are important in many processes of the body. Rodents, such as mice and rats, are typical animal models used to research the impact of micronutrients in their diets on the function of the organism. Copper (Cu) and zinc (Zn) are micronutrients engaged in an organisms’ physiological and metabolic processes. They are given to laboratory rats with food (in the recommended doses of Cu: 4 mg/kg and Zn: 11 mg/kg), but they also can be given in water (Open Formula Rat and Mouse Diets–Minerals. National Institutes of Health Feed & Bedding Standards & Specifications) [1].

Copper is essential for respiration, the removal of free radicals, the production of energy, the development of connective tissues, the metabolism of iron and oxygen, and the maturation of extracellular matrix and neuropeptides, as well as neuroendocrine signaling [2]. To maintain muscle contraction, biosynthesis of peptide hormones, oxidative stress protection and other essential functions, cardiac tissue exhibits a significantly higher copper requirement than other tissues [3]. Following ingestion of a large amount, copper begins to accumulate in the liver, preventing it from detoxifying the body’s elevated copper levels. This negatively impacts the nervous system, adrenal function, reproductive system, and connective tissue. If a considerable amount is consumed all at once, it results in extreme nausea, cramps, and vomiting, and in serious situations, convulsions or paralysis may cause death [4]. Excessive copper levels have the potential to induce oxidative stress, a critical contributor to the initiation and advancement of type 2 diabetes mellitus [5]. Moreover, copper deficiency plays an important role in the unspecific manifestations of hematologic, neurologic, immunologic, dermatologic, cardiovascular, and skeletal defects [6]. Majewski et al. measured blood plasma copper, zinc and ceruloplasmin (Cp) content in Wistar rats fed a copper-deficient diet. The obtained results pointed to the decreased blood plasma copper/zinc ratio and increased Cu/Cp due to increased copper and Cp. In the same study, isolated aortic rigs from a copper-deficient diet exhibited an increased contractile response to high KCl (75 mM), endothelin-1 and prostaglandin F2-alpha [7].

The duodenum and small intestine are mostly responsible for the absorption of dietary copper [8], and its uptake into intestinal epithelial cells is primarily mediated by copper transport protein 1 (CTR1), which is localized on the apical side of enterocytes [9]. Copper is released into the bloodstream and binds to soluble chaperones, such as albumin, transcuprein, histidines, and macroglobulins, following absorption via the gastrointestinal system. Copper is taken up by the hepatocytes via CTR1 upon reaching the liver. It is subsequently supplied by copper chaperones to specific proteins or chelated by metallothionein (MT) for storage inside the cytoplasm. Principal copper chaperones transfer copper ions from the liver back into circulation, where they reassociate with soluble chaperones and are delivered to particular tissues and organs [10]. Copper is stored mainly in the liver, and an excess amount is removed through biliary excretion through the stool. Other routes of elimination, such as urine, perspiration, and menstruation, play a limited role in its excretion [11].

Zinc as a microelement is essential for healthy cellular activity, which includes transcription, protein synthesis, RNA and DNA synthesis, DNA replication, cell division, growth, and differentiation. It also plays a role in the endocrine and immunological systems. Zinc affects hormone synthesis, receptor function, T4 to T3 conversion, and carrier protein formation. Zinc boosts growth hormone production and is abundant in pancreatic tissue, regulating insulin action [12]. Zinc also exerts a stimulatory effect on the metabolic pathway known as glycolysis. Furthermore, it acts as an inhibitor of gluconeogenesis, a process involved in the synthesis of glucose. Additionally, zinc is involved in facilitating the transportation of glucose in adipocytes. Alongside with insulin, zinc also plays a role in the suppression of glucagon release in the presence of elevated glucose levels [13]. The administration of zinc supplements leads to a notable reduction in the total cholesterol, low-density lipoprotein cholesterol, and triglyceride levels, while concurrently promoting an increase in high-density lipoprotein cholesterol among patients [13]. Obese people have low zinc and high leptin, indicating a crucial link between the two. Zinc enzymes synthesize melatonin, whereas melatonin regulates intestinal zinc absorption. The zinc-dependent enzyme 5α-reductase converts testosterone to dihydrotestosterone [12]. Zinc is essential for neutrophil and NK cell growth and activity. Although zinc is present in many tissues, the largest proportion is found in the testes, muscle, liver, bone and brain. It is widely distributed in synaptic vesicles and is crucial for memory and learning. Moreover, zinc serves as a cofactor for more than 2000 transcription factors and 1000 enzymatic processes [14].

The majority of acute zinc overload symptoms are only located in the gastrointestinal tract. Acute renal injury, pancreatic injury, liver failure, and hemodynamic instability are further symptoms of severe zinc poisoning. Chronic zinc poisoning can result in iron deficiency anemia that is resistant to iron supplementation. Interestingly, zinc overload can also cause symptoms similar to copper deficiency [15].

Zinc insufficiency affects macrophages, phagocytosis, intracellular killing, and cytokines, while hindering T and B cell development [16]. Low dietary intake of this mineral can manifest as growth impairment, inflammation, sexual dysfunction, gastrointestinal issues, or skin involvement [17]. Specific transporters found in the apical membrane of enterocytes absorb zinc in the duodenum and the first part of the jejunum, as in the case of copper [18]. The absorption of zinc is facilitated by citric acid and blocked by iron, fiber, and phytate, a zinc chelator [19]. Zinc is bound to MT inside the cells of the enterocytes or sent to the liver through the portal vein. The MT-bound fractions are later returned to the intestine via enterocyte shedding [18]. Zinc intracellular and plasma concentrations are constant, as it is regulated by several processes such as intestinal absorption or excretion, urine excretion, and cellular retention [20]. 

## 2. Effects of Copper and Zinc on Heart Functioning

Copper is delivered to organs and tissues, including the heart, after entering the circulation via attachment to plasma proteins such as ceruloplasmin (CP), trans copper protein, albumin, and other plasma proteins [21]. Copper exists in two distinct ionic states, namely Cu(I) (referred to as cuprous ion or the reduced form) and Cu(II) (known as copper ion or the oxidized form). These two forms of copper are involved in the enzymatic modulation of various physiological processes within the cell [22]. Cu(II) affects the interaction between growth factors and cell membrane receptors. Once Cu(II) enters cell membranes, metalloreductases will decrease it. Then, monovalent Cu(I) can alter the structure of proteins or phosphorylation status, hence modifying the activation status of receptors for growth factors in the plasma membrane [23]. Cu(I) controls the redox equilibrium in numerous organelles in the cytoplasm and modulates kinase activity directly by altering the structure of phosphatase. Cu(I) influences the expression of genes and subsequent protein synthesis in the nucleus by binding transcription factors [23]. Copper ions undergo reduction and generate hydroxyl radicals (^•^OH) that initiate reactions with DNA and lipids, inducing DNA impairment and lipid peroxidation in the cardiovascular system, correspondingly [23].

In some studies, the time course of a high-iron diet has been linked to anemia, tissue copper depletion, hypopigmentation, and serum CP activity suppression. After mice were supplemented with copper, several physiological disturbances were partially or completely corrected, including the observed anemia, decreased tissue copper concentrations, pigmentation defects, cardiac hypertrophy, and decreased serum CP activity [24]. Excessive copper had an impact on cardiac troponin I (c-TnI) and N-terminal forebrain natriuretic peptide (NT-pro-BNP) content in mouse serum, as the levels of these factors tended to rise in copper-laden mouse organisms in vivo [25]. In addition, mice who were given an excessive amount of copper per os had significantly increased cases of cardiac fibrosis, which led to even more severe myocardial damage. Apoptosis was eventually caused by an excess of copper because it altered the ratio of apoptotic components and ultimately caused cell death [25]. Furthermore, copper concentrations in the serum and myocardium of diabetic rodents were significantly elevated. This demonstrated that diabetes may facilitate copper excess, which may directly affect cardiomyocyte function in diabetic individuals. Cardiomyocyte mortality is followed by cardiomyocyte hypertrophy, fibroblast proliferation, and an increase in the extracellular matrix, resulting in myocardial remodeling and cardiac dysfunction in diabetic cardiomyopathy [26]. Additionally, copper is involved in vascular changes in the heart. Kunutsor et al. showed that a higher serum copper level increases the risk of atherosclerotic heart disease by regulating lipid metabolism, low-density lipoprotein oxidation, and inflammatory response [27]. Cuproptosis, a recently discovered copper ion-dependent programmed cell death, could thus be implicated in the development of coronary artery disease, and the genes associated with cuproptosis may serve as marker genes for coronary artery disease [28].

Albumins are responsible for up to 98% of the exchangeable component of Zn^2+^ found in plasma, which accounts for approximately 75–80% of the plasma Zn^2+^ that is related to albumin [29]. Albumin in serum has been shown to be an effective “zinc buffer” for the extracellular environment. Therefore, serum albumin not only regulates the uptake of Zn^2+^ into cells such as endothelium and erythrocytes, but it also protects blood cells and endothelial cells lining blood arteries from the potentially harmful quantities of Zn^2+^ in plasma [30]. Quick absorption of zinc ions occurs in endothelial cells, most likely by the endocytosis of zinc that is linked to albumin [31]. Albumin in the serum is also involved in the management of Cu^2+^ in animals, which contributes to the regulation of the levels of this metal in blood that are physiologically active [32]. Zrt-/Irt-like protein (ZIP) are zinc transporters, which increase cytoplasmic zinc levels by importing zinc, while ZnTs are zinc transporters that reduce cytoplasmic zinc levels via zinc export. Both are present in mammals [33]. Thokala et al. demonstrated that zinc supplementation increased ZnT1, ZnT2 mRNA while decreasing ZnT5, ZIP1, ZIP2, ZIP3, ZIP7, ZIP9, and ZIP10 mRNA. Zinc’s negative regulation of ZIPs appears to protect cells from zinc toxicity by decreasing zinc influx. Similarly, under zinc-deficient conditions, ZnTs expression appears to be regulated to prevent zinc loss from the intracellular environment [34]. There is a correlation between zinc intake or plasma/serum zinc levels and the future occurrence of cardiovascular disease. A higher serum zinc level was connected with a decreased risk of cardiovascular disease [35]. In addition, recent discoveries in cardiac biology and pathophysiology have shown the critical role that disruptions in zinc homeostasis play in the development of myocardial ischemia or reperfusion injury, as well as the role that zinc signaling plays in the development of cardio-protection against ischemia/reperfusion injury [36].

In a rat model of ischemia/reperfusion, in which isolated rat hearts were perfused using the Langendorff technique, the cardioprotective significance of intracellular zinc was established. Particularly, the results demonstrated reduced zinc levels in ischemic/reperfusion with zinc ionophore pyrithione treatment during reperfusion to increase myocardial healing to nearly 100% and decrease arrhythmias by more than twice [37]. Moreover, ZnT1 is implicated in the electrophysiological effects of zinc on the heart, and individuals with atrial fibrillation exhibit elevated ZnT1 expression [38]. One month after treatment, morphological analysis of organs and tissues in rats treated with zinc succinate at a single intragastric dose of 100 mg/kg compared to animals in the control group revealed toxic and dystrophic alterations in the brain, heart, lungs, and liver [39].

In addition, the protective effect of zinc compounds on the heart has also been studied. Wu et al. studied the cardiotoxicity of doxorubicin, and the protective effect on the myocardial tissue of Zn(II)-curcumin (ZnCM) was proved [40]. ZnCM assisted in the preservation of cardiac structure in rodents treated with doxorubicin. Thus, ZnCM could be an innovative candidate for preventing or reducing doxorubicin-induced cardiotoxicity in cancer patients.

## 3. Effects of Copper and Zinc on Vasculature

Recently, only a few studies were performed examining the effects of copper and zinc on vascular contractility, especially their excess. Wang et al. investigated direct effects of copper on the vasoactivity of rat mesenteric artery in vitro. Copper was administered at various concentrations to the mesenteric artery previously contracted by noradrenaline (ex vivo studies). Copper induced a dose-dependent vasodilation of the mesenteric rings. However, this was substantially inhibited when the arteries were pretreated with an endothelial nitric oxide synthase (eNOS) inhibitor L-NAME [41]. However, according to the preceding research, extended incubation of aortic rings with Cu^2+^ at concentrations below 1 µM resulted in the impairment of a relaxing response to acetylcholine while having no impact on the response elicited by the NO-donors’ isosorbide dinitrate and 1,1-diethyl-2-hydroxy-2-nitroso-hydrazine sodium. This study proposed that the vasorelaxation of rat aorta induced by acetylcholine is solely dependent on the release of NO, and that the administration of Cu^2+^ had a discernible impact on NO-mediated vasorelaxation [42]. Furthermore, Lamb et al. conducted an investigation on the relaxation of carotid artery rings in response to calcium ionophore (which facilitates copper transport). The results indicated that the relaxation was more pronounced in arteries obtained from rabbits supplemented with copper [43]. Majewski et al. studied the impact of dietary resveratrol on young Wistar rats fed for eight weeks with a copper deficient diet. This study demonstrated that resveratrol supplementation modulated antioxidant status, which potentiated both vascular contraction to noradrenaline and relaxation to acetylcholine [44].

Kusleikaite et al. demonstrated that the impact of a 48-day stress immobilization is significant in the development of endothelial dysfunction. This research reveals that the relaxation of smooth muscles in the thoracic aorta of rabbits lacking zinc supplementation was significantly lower in response to acetylcholine, as compared to those rabbits that received zinc supplementation. Moreover, it was observed that in rabbits subjected to stress immobilization without zinc supplementation, the endothelium dependent relaxation of smooth muscles was significantly reduced compared to the control rabbits, across all concentrations of acetylcholine. The potential mechanism underlying the relaxation of smooth muscle in rabbits administered with zinc may involve the release of NO and endothelium-derived hyperpolarizing factors in response to acetylcholine stimulation of the vascular wall [45]. Furthermore, the study conducted by Betrie et al. established the involvement of zinc in the regulation of vascular tone. The authors provided evidence that an increase in intracellular zinc levels leads to vasorelaxation through its impact on multiple pathways, including the signaling of calcitonin gene-related peptide from perivascular sensory nerves mediated by transient receptor potential cation channel subfamily A (ankyrin) member 1, the synthesis of dilatory prostanoids by endothelial cells, and the inhibition of voltage-gated calcium channels in smooth muscles [46].

Yanagisawa et al. investigated the impact of excessive zinc consumption on kidney function and blood pressure. The findings revealed that the intake of excess zinc led to a decline in renal function, which was attributed to a heightened activity of exogenous superoxide radicals (^•^O_2_^−^). The authors concluded that excessive consumption of zinc may play a crucial role in increasing systemic blood pressure and decreasing renal function due to the oxidative stress induced by exogenous superoxide radicals [47].

The in vivo and in vitro studies are summarized in Table 1 and Table 2.

## 4. The Impact of Nanoparticles of Copper and Zinc on the Cardiovascular System

Different metals and metal oxides in the form of nanoparticles (NPs) were recently studied as food additives due to their specific nano-scale properties (Table 3) [48,49,50]. Tousson et al. showed rats who consumed copper oxide nanoparticles (CuO-NPs) experienced a statistically significant reduction in body weight gain. According to increases in serum creatine kinase-MB, creatine kinase enzyme, serum lactate dehydrogenase, serum myoglobin, aspartate aminotransferase and alkaline phosphatase, CuO-NPs induced cardiac dysfunction and toxicity. The increase in cardiac enzymes could be attributed to the CuO-NPs’ emission of free radicals. CuO-NPs induced severe cardiac injury, characterized by myocardial hypertrophy and severe focal necrosis of cardiac myocytes accompanied by an infiltration of inflammatory cells [51]. According to Cholewińska et al. the substitution of copper carbonate (ionic form of copper) with metal nanoparticles in the food consumed by rats could worsen the adverse effects caused by hypertension on the heart, liver, and intestines [48]. Moreover, the study conducted by Majewski et al. demonstrated that the consumption of copper nanoparticles (CuNPs) leads to elevated levels of oxidative stress. This, in turn, affects the process of vascular relaxation by involving 20-hydroxyeicosatetraenoic acid (20-HETE) and the thromboxane-A2 receptors [49]. Prior studies have indicated that the ingestion of nano copper can elevate lipid peroxidation, promote vasodilation, and augment NO production via heightened iNOS expression [52]. This modulation of the oxidative stress is attributed mainly to the NPs rather than copper itself [53]. Furthermore, CuNPs enhanced vascular contraction induced by prostaglandin F2-alpha and decreased the blood plasma Cu–Zn ratio in Wistar rats [7]. Our previous studies already established that oxidative stress when overwhelming the antioxidant defense mechanisms induces functional changes in arteries [54]. Interestingly, administration of fish oil together with CuNPs modified the superoxide dismutase, catalase, and decreased copper concentration in the blood. Moreover, fish oil elicited a favorable response in the vascular system and mitigated noradrenaline-induced aortic contraction (in vitro) in rats that received ionic copper supplementation, whereas no such effect was observed in rats that received CuNPs [55]. The study conducted by Sharma et al. demonstrated that the administration of a low dose of CuNPs and physical exercise training, either individually or in combination, resulted in the inhibition of glycogen synthase kinase 3β expression. This was achieved by up-regulating the phosphorylation pathway, which further contributed to the reduction of oxidative stress, release of inflammatory mediators, occurrence of apoptotic events, and an increase in the serum bioavailability of NO. These effects were observed in rats with ischemia/reperfusion-induced myocardial injury [56].

According to another study, human umbilical vein endothelial cells (HUVECs) were found to be susceptible to CuO-NPs, which led to DNA damage and cell death in these cells (in vitro). In addition, oxidative stress and activation of the p38 mitogen-activated protein kinase pathway were both enhanced in HUVECs by CuO-NPs [57].

The toxicity of excess zinc nanoparticles was also observed in vitro in rat cardiomyoblasts, where it was detected in the mitochondria and endoplasmic reticulum along with the disruption of the cytoplasmic membrane and collapsed nuclei with a low density of nuclear material [58]. In addition, it was discovered that ZnO-NPs were associated with the inhibition of cell proliferation or loss of cell viability, as well as mitochondrial damage and cell mortality. After 48 h of treatment with 10 μg/cm^2^ ZnO-NPs, a substantial decrease in cell proliferation was observed. The number of necrotic cells increased to 5 μg/cm^2^ [58]. Moreover, ZnO-NPs (10 μg/cm^2^) substantially decreased the expression of intracellular troponin I and atrial natriuretic peptide by 27% and 20%, respectively [58].

Furthermore, Asri-Rezaei et al. showed that pharmacological effects of zinc nanoparticles are dose-dependent. In diabetic rats, a lower dose of 1 mg/kg had minimal to negligible beneficial effects, while a middle dose of 3 mg/kg induced significant cardioprotective action. In contrast, it was observed that the administration of the maximal dose (10 mg/kg) elicited unfavorable outcomes and exacerbated complications associated with diabetes [59].

Yan et al. proved that rats fed 5 mg/kg ZnO-NPs exhibited a distended vessel wall, damaged smooth endometrial endothelial cells, and had a discontinuous or fragmented intimal surface. ZnO-NP exposure may induce atherosclerotic alterations via direct and indirect mechanisms [60]. Chen et al. demonstrated the essential role that ZnO-NPs play in increasing the permeability of blood vessels in the lungs of mice by studying the influx of immune cells into the lung tissue in vivo. In addition, they confirmed that this was caused by a decrease in the expression of certain proteins that create intercellular junctions through in vitro research conducted on human endotheliocytes [61]. 

Liang et al. found that ZnO-NPs have an apoptotic role in human aortic endothelial cells (HAECs) [62]. Poier and Tada-Oikawa supported the aforementioned results in rats. They found that doses of ZnO-NPs above 20 µg/mL reduced cell viability, increased apoptosis [63], as well as contributed to the formation of new blood vessels [64]. Additionally, it has been demonstrated that the duration of exposure to ZnO-NPs is crucial [62,63].

The in vivo and in vitro studies on metal and metal oxide nanoparticles are summarized in Table 3. 

**Table 3:** The impact of copper and zinc nanoparticles on the cardiovascular system.

Compound	Dose	Duration of Study/Incubation Time	Experimental Model	Effect on the Cardiovascular System	References
CuNPs (40 nm)	6.5 mg/kg CuCO_3_ or CuNPs (100% replacement) or 3.25 mg/kg CuCO_3_ plus 3.25 mg/kg CuNPs (50% replacement)	8 weeks in food	Wistar rats	CuNPs enhanced vascular contraction induced by prostaglandin F2-alpha and decreased the blood plasma Cu-Zn ratio	[7]
			WKY and SHR	CuNPs exacerbated the negative changes induced by hypertension in the heart, liver, and intestines	[48]
			young WKY rats	CuNPs in both doses modified vasodilation through the vasoconstrictor 20-HETE and the TP receptors	[49]
			young Wistar rats	CuNPs influenced oxidative stress, which further modified the vascular response	[52]
			Wistar rats	interaction between fish oil and CuNPs may follow the replacement of CuCO_3_ with CuNPs	[55]
CuO-NPs	100 mg/kg body weight/day	2 to 4 weeks	rats	induced cardiac dysfunction and toxicity	[51]
CuO-NPs	0, 10, 20, or 40 μg/mL	0–24 h	HUVECs	DNA damage and cell death, enhanced oxidative stress	[57]
ZnO-NPs	2.5, 5, 10, and 20 μg/cm^2^	24, 48, 72 h	rats cardiomyoblasts	severe damage to cardiomyocytes	[58]
ZnO-NPs	0, 1.25, 2.5, and 5.0 mg/kg	12 weeks	rats	atherosclerotic alterations	[60]
ZnO-NPs	0, 3, 10 or 30 µg/mouse	4 weeks	mice	increased permeability of blood vessels in the lungs	[61]
ZnO-NPs	8–50 μg/mL	12 and 24 h	HAECs	apoptotic role	[62]
ZnO-NPs	20, 25, 30, 35, 40, 45 and 50 µg/mL	24 h	HUVEC	above 20 µg/mL increased apoptosis	[63]

## 5. Conclusions

Copper and zinc are essential nutrients for the proper functioning of the body. The significance of their role in preserving the body’s oxidoreductive equilibrium and safeguarding cells against free radicals is noteworthy. The correlation between serum copper and zinc concentrations and cardiovascular disease was observed. However, the general population lacks a precise diagnostic approach, and the specific concentrations of these metals in distinct cardiovascular diseases remain undetermined, necessitating further investigation. Both a deficiency and an excess of either copper or zinc can have unfavorable effects on one’s health. The limited number of experimental studies pertaining to the effects of excess copper and zinc on the cardiovascular system warrants further scientific investigation on this subject matter. Hence, it appears reasonable to pursue additional research on excess dietary copper and zinc.

## Figures and Tables

**Table 1:** In vivo studies.

Compound	Dose	Method of Administration	Duration of Study	Experimental Model	The Effect	References
CuSO_4_ pentahydrate	20 or 200 mg/L of copper	per os in water	2 weeks	mice	compensation for disorders caused by a high-iron diet	[24]
CuCl_2_	1.08 g/kg	per os in water	8 to 14 weeks	mice	increase in markers of myocardial damage, cardiac fibrosis	[25]
Zn pyrithione	35 mg/kg	per os in diet	1 week	male Sprague Dawley rats	increased myocardial healing and decreased arrhythmias during reperfusion	[37]
Zn succinate	100 mg/kg	per os in water	1 month	male Wistar rats	toxic and dystrophic alterations in the heart	[39]
Zn(II)–curcumin	25, 50 and 100 mg/kg	by oral gavage	2 weeks	male Sprague Dawley rats	protects the heart of rats from damage during treatment with doxorubicin	[40]
Zn(II) acetate	0.3 mg/kg	per os in diet	48 days	chinchilla male rabbits	defends endothelial cell ultrastructure and function	[45]
Zn	1.1, 11 and 44 mg/day	per os in diet	4 weeks	male Sprague Dawley rats	decline in renal function, increased blood pressure induced by superoxide radicals	[47]

**Table 2:** In vitro/ex vivo studies.

Compound	Dose	Incubation Time	Experimental Model	The Effect	References
CuCl_2_ and copper ionophore	10 μM	48 h	mice cardiomyocytes	diabetic cardiomyopathy may be caused by cuproptosis	[26]
CuCl_2_	10–200 μM	10 min	rats mesenteric arteries	dilation of the mesenteric artery, inhibited by L-NAME	[41]
CuSO_4_	2 μM	3–24 h	rat aorta	impairment of the relaxing response to acetylcholine	[42]
CuSO_4_	10 mM	2 h	rabbit smooth muscle cells	improved relaxation of arteries	[43]

## Data Availability

Not applicable.
